# Supplementary material for: An actor-model framework for visual sensory encoding
Source: Nat Commun. 2024 Jan 27;15:808. doi: 10.1038/s41467-024-45105-5 (PMC10821921; doi:10.1038/s41467-024-45105-5)
Supplement: Supplementary file 1 — Supplementary Information [file 41467_2024_45105_MOESM1_ESM.pdf]

An actor-model framework for visual sensory encoding

Franklin Leong[1], Babak Rahmani[2,+], Demetri Psaltis[3], Christophe Moser[2] and Diego Ghezzi[1,4,*]

[1] Medtronic Chair in Neuroengineering, Center for Neuroprosthetics and Institute of Bioengineering, School of Engineering, École Polytechnique Fédérale de Lausanne, Geneva, Switzerland

[2] Laboratory of Applied Photonics Devices, Institute of Electrical and Micro Engineering, School of Engineering, École Polytechnique Fédérale de Lausanne, Lausanne, Switzerland

[3] Optics Laboratory, Institute of Electrical and Micro Engineering, School of Engineering, École Polytechnique Fédérale de Lausanne, Lausanne, Switzerland

[4] Ophthalmic and Neural Technologies Laboratory, Department of Ophthalmology, University of Lausanne, Hôpital ophtalmique Jules-Gonin, Fondation Asile des Aveugles, Lausanne, Switzerland

[+] Current affiliation: Microsoft Research, Cambridge, UK

[*] Corresponding author: info@ghezzilab.org

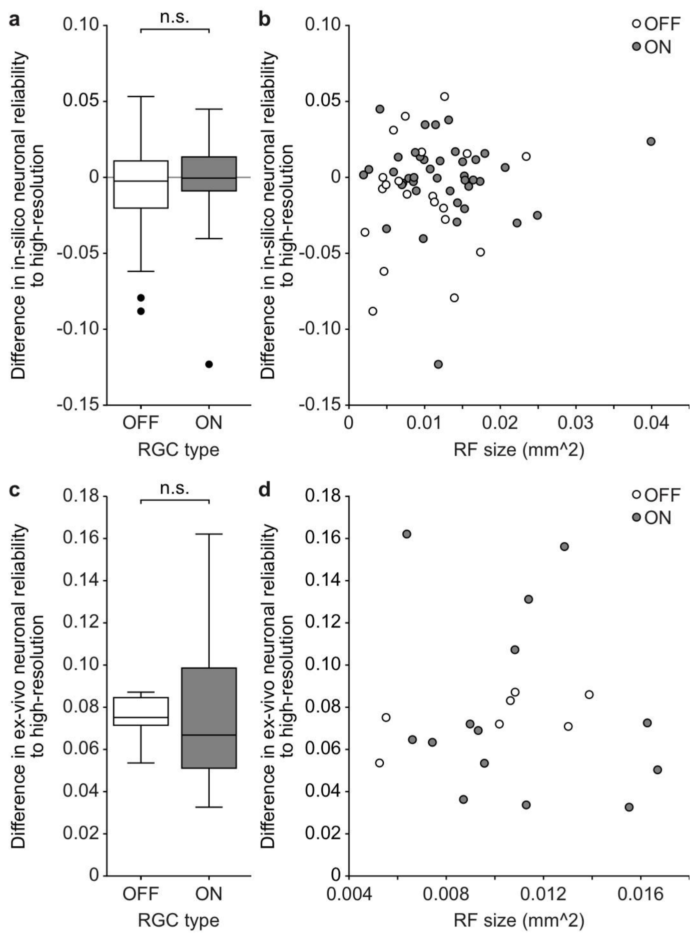

Supplementary Figure 1. Neuronal reliability is neither dependent on the cell type nor the RF size. a, Difference in in-silico neuronal reliability of actor downsampled images to high-resolution images as a function of the cell type (n = 60 modeled neurons; two-tailed Mann-Whitney U test, p = 0.3845 reported as n.s.). The box spans from the 25th to the 75th percentiles, the line is the median, and the whiskers are 1.5 times the interquartile range. The black dots indicate outliers. **b**, Difference in in-silico neuronal reliability of actor downsampled images to high-resolution images as a function of the RF size (n = 60 modeled neurons; Pearson correlation coefficient r = 0.0713, p = 0.5885). **c**, Difference in ex-vivo neuronal reliability of actor downsampled images to high-resolution images (n = 21 RGCs from N = 8 retinas; two-tailed Mann-Whitney U test, p = 0.4003 reported as n.s.). The box spans from the 25th to the 75th percentiles, the line is the median, and the whiskers are 1.5 times the interquartile range. **d**, Difference in ex-vivo neuronal reliability of actor downsampled images to high-resolution images as a function of the RF size (n = 21 RGCs from N = 8 retinas; Pearson correlation coefficient r = -0.0838, p = 0.7181).

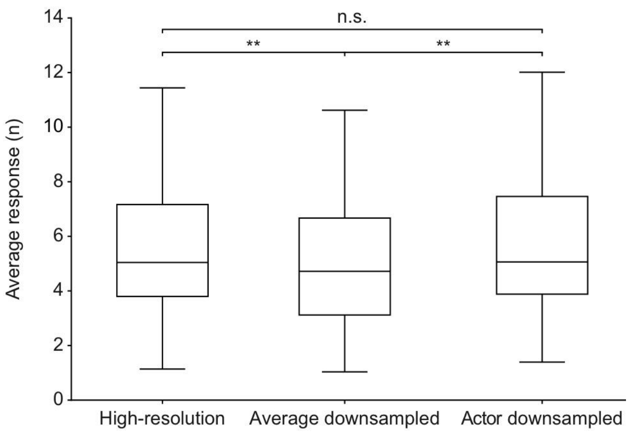

Supplementary Figure 2. **Response of RGCs in ex-vivo experiment.** Quantification of the average response of RGCs upon presentation of high-resolution, average downsampled and actor downsampled images (n = 21 RGCs from N = 8 retinas). The response is the sum of the number of spikes occurring during image projection (400-ms window). The box spans from the 25th to the 75th percentiles, the line is the median, and the whiskers are 1.5 times the interquartile range. Friedman test: $p < 0.0001$. Nemenyi post-hoc tests: average downsampled versus high-resolution ($p = 0.001$, reported as **), average downsampled versus actor downsampled ($p = 0.001$, reported as **), actor downsampled versus high-resolution ($p = 0.5398$, reported as n.s.).

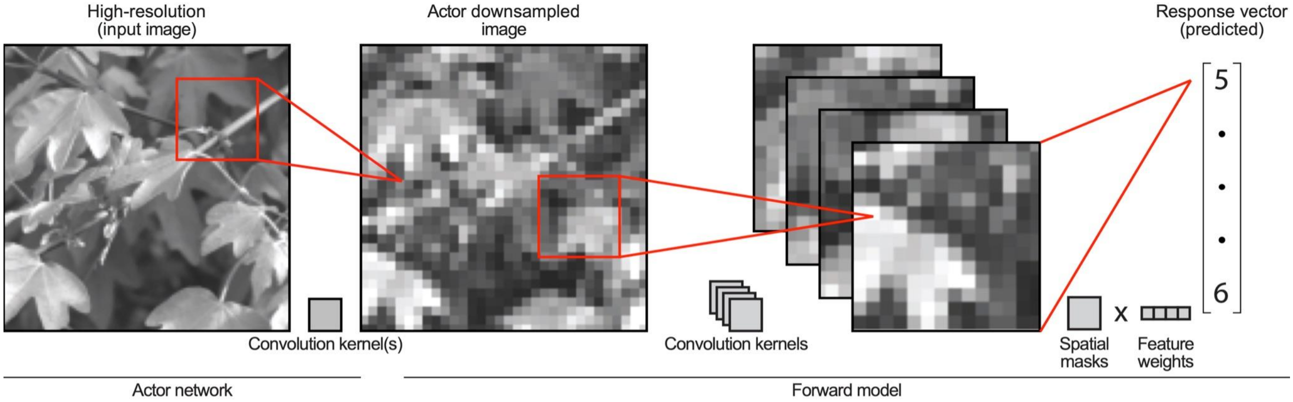

Supplementary Figure 3. Network architecture. The forward model comprises a layer of convolution kernels, followed by the readout layer, which is decomposed into spatial masks and feature weights. The actor network is prepended to the trained forward model and consists of a layer of convolution kernels. The schematic illustrates the flow when a single image is passed as input through the framework to generate the predicted response vector of the neurons to one image.
